# The effect of *hippophae rhamnoides* extract on oral mucositis induced in rats with methotrexate

**DOI:** 10.1590/1678-775720160139

**Published:** 2016

**Authors:** Ozan Kuduban, Muhammed Recai Mazlumoglu, Selma Denktas Kuduban, Ertugrul Erhan, Nihal Cetin, Osman Kukula, Oguzhan Yarali, Ferda Keskin Cimen, Murat Cankaya

**Affiliations:** 1 Erzurum Education and Research Hospital, Ear Nose and Throat Head&Neck Surgery Clinic, Erzurum, Turkey.; 2 Hinis State Hospital, Department of Otorhinolaryngology, Erzurum, Turkey.; 3 Palandoken State Hospital, Department of Plastic Reconstructive and Esthetic Surgery, Erzurum, Turkey.; 4 Erzincan University, Faculty of Medicine, Department of Otorhinolaryngology, Erzincan, Turkey.; 5 Erzincan University, Faculty of Medicine, Department of Pharmacology, Erzincan, Turkey.; 6 Ondokuzmayis University, Faculty of Medicine, Department of Pharmacology, Samsun, Turkey.; 7 Erzurum Training and Research Hospital, Department of Medical Genetics, Erzurum, Turkey.; 8 Mengucek Gazi Education and Research Hospital, Department of Pathology, Erzincan, Turkey.; 9 Erzincan University, Faculty of Arts and Sciences, Department of Biology, Erzincan, Turkey.

**Keywords:** Gene expression, Hippophae rhamnoides, Methotrexate, Oral mucositis, Rats

## Abstract

**Objective:**

To investigate the effect of HRE (*Hippophae rhamnoides* extract) on oral mucositis induced in rats with MTX.

**Material and Methods:**

Experimental animals were divided into groups as healthy (HG), HRE+MTX (HMTX), and control group, which received MTX (MTXC). HMTX group received 50 mg/kg HRE while MTXC and HG groups received equivolume distilled water with gavage once a day. After one hour of HRE and distilled water administration, HMTX and MTXC groups received a single dose of oral MTX 5 mg/ kg. This procedure was repeated for one month.

**Results:**

The levels of MDA, IL-1β, and TNF-α were found to be significantly higher in the cheek, lower lip, and tongue tissue of the animals receiving MTX, compared with HG and HMTX groups; however, these parameters were lower in the cheek and low lip tissue, and a milder damage ocurred in these tissues, compared with the tongue tissue in MTXC group. No histopathologic damage was observed in the cheek, lower lip, and tongue tissues of the rats treated with HRE.

**Conclusion:**

This findings indicate that HRE as a natural product is an important advantage compared with synthetic drugs for prophylaxis of oral mucositis developed due to MTX.

## INTRODUCTION

Methotrexate (MTX) is an antiproliferative folic acid antagonist used in the treatment of various cancers and chronic inflammatory diseases. Low-dose MTX is used to treat inflammatory diseases and high doses are used to treat malignancies^2^. However, high-dose MTX cause serious adverse effects^29^. MTX and some antineoplastic drugs destruct the proliferating cells of the mucosal layer, creating damage in the oral tissues^14^. This side effect, known as oral mucositis, occurs in 40% of patients receiving chemotherapy^12^. Mucositis reportedly consists of steps beginning with the formation of reactive oxygen species (ROS), progressing with the release of proinflammatory cytokines, such as tumor necrosis factor (TNF-α) and interleukin-1 beta (IL-1 β), resulting in mucosal damage, infection, and cell death^21^. Oral mucositis causes either discontinuation or reduced chemotherapy dose^19,28^. Therefore, numerous studies have been conducted about protection against mucositis, but there are no specific medications in clinical practice for the treatment of mucositis. MTX has been reported to decrease the glutathione level and to significantly increase levels of myeloperoxidase (MPO), malondialdehyde (MDA), interleukin 1 beta (IL-1β), and tumor necrosis factor (TNF-α), which are indicators of inflammatory response in the gastrointestinal system^1,16^. This information from the literature indicates that MTX may also damage the oral mucosa, showing antioxidant, antiinflammatory, antiulcer, and antimicrobial effects, and inhibiting the production of proinflammatory cytokines. *Hippophae rhamnoides* fruit extract (HRE), tested in this study against MTX oral mucositis, showed antioxidant, antiulcerogenic, antiinflammatory, antimicrobial, and proinflammatory cytokine antagonist properties^18,27,31^. *Hippophae rhamnoides L.* plant, member of the *Eleagnaceae* family, contains carotenoids (α, β, γ), riboflavin, vitamin C, tocopherol, tocotrienol, folic acid, tannin, and fatty acids^18,31^. No studies on the protective effect of HRE against oral mucositis induced by MTX were found in the literature screening. Therefore, the objective of this study is to investigate and evaluate the effect of HRE on oral mucositis induced in rats with MTX through biochemical, gene expression, and histopathologic examinations.

## MATERIAL AND METHODS

### Animals

Experimental animals were obtained from Atatürk University Medical Experimental Application and Research Center. A total of 21 rats weighing 224-232 g were used. Before the experiment, animals were doused and fed as groups (n-7) in the pharmacology laboratory at normal room temperature (22°C). The study was conducted in Ataturk University Experimental Studies and Research Center, Erzurum. The experimental procedure was approved by the Committee for Animal Research of Ataturk University, Erzurum. This study was carried out in accordance with international guidelines on the ethical use of animals. (Ethics Committee Number: 23.10.2015/168).

### Chemical agents

The chemical agents used in the experiment were methotrexate supplied from Med-İlaç-Turkey, Thiopental sodium from I.E. Ulagay-Turkey, and *Hippophea rhamnoides* extract from Karen Bilim-Turkey.

### The experiment

Experimental animals were divided into groups as healthy (HG), HRE+MTX (HMTX), and control group, which received MTX (MTXC). HMTX group of rats (n-7) was given 50 mg/kg HRE while MTXC (n-7) and HG (n-7) groups were given equivolume distilled water with gavage once a day. After one hour of HRE and distilled water administration, HMTX and MTXC groups received a single dose of oral MTX 5 mg/kg. This procedure was repeated for one month. At the end of this period, all animals were sacrificed with high-dose anesthesia. Then the amounts of MDA and tGSH were determined in the removed cheek, lower lip, and tongue tissues. In addition, IL-1β and TNF-α gene expressions were measured, and all the tissues were histopathologically studied.

### Biochemical analyses

#### MDA analysis

According to the method defined by Ohkawa, et al.^22^ (1979), MDA forms a pink complex with thiobarbituric acid (TBA) at 95°C, which can be measured using spectrophotometry at a wavelength of 532 nm^22^. The volume of 0.1 mL homogenat was added to a solution containing 0.1 mL of 8.1% sodium dodecyl sulphate (SDS), 1.5 mL of 20% acetic acid (Merck, Darmstadt, Hessen, Germany) 1.5 mL of 0.9% TBA (Sigma-Aldrich, Steinheim, Nordrhein-Westfalen, Germany), and 0.3 mL dH_2_O. The mixture was incubated at 95°C for 1 h. Upon cooling, 5 mL of n-butanol: pyridine (v/v, 15:1, Merck, Darmstadt, Hessen, Germany) was added. The mixture was vortexed for 1 min and centrifuged for 30 min at 4000 rpm. The absorbance of the 0.15 mL supernatant was measured at 532 nm by spectrophotometry. The Standard curve was obtained by using 1,1,3,3-tetramethoxypro pane (Sigma-Aldrich, Steinheim, Nordrhein-Westfalen, Germany).

#### tGSH analysis

According to the method defined by Sedlak, et al.^25^ (1968), DTNB [5,5'-dithiobis (2-nitrobenzoic acid)] disulfite is chromogenic in the medium, and DTNB is reduced easily by sulfhydryl groups. The yellow color produced during the reduction is measured by spectrophotometry at 412 nm^25^. For measurement, cocktail solution [5.85 mL 100 mM Na-Fosfat buffer, 2.8 mL 1 mM DTNB (Sigma-Aldrich, Steinheim, Nordrhein-Westfalen, Germany), 3.75 mL 1 mM NADPH (Sigma-Aldrich, Steinheim, Nordrhein-Westfalen, Germany), and 80 μL 625 U/L Glutathione reductase (Sigma-Aldrich, Steinheim, Nordrhein-Westfalen, Germany)] was prepared. Before measurement, 0.1 mL metaphosphoric acid (Sigma-Aldrich, Steinheim, Nordrhein-Westfalen, Germany) was added onto 0.1 mL homogenate and centrifuged for 2 min at 2000 rpm for deproteinization. The volume of 0.15 mL cocktail solution was added onto 50 μL of supernatant. The Standard curve was obtained using GSSG (Sigma-Aldrich, Steinheim, Nordrhein-Westfalen, Germany).

### Gene expression of IL-1β and TNF-α

**RNA isolation:** RNA was isolated from the homogenizated oral tissue samples using the Roche Magna Pure Compact LC device (Roche Diagnostics GmbH, Meinheim, Germany) with MagNA MagNA Pure LC RNA Kit (Roche Diagnostics GmbH, Mannheim, Germany). The quantity and quality of the isolated RNA were assessed with a nucleic acid measurement device (Maestro, Nano, Nucleotest Bio Ltd. 1038 Budapest, Hungary). Fifty μL of RNA samples were stored at −80°C.

cDNA synthesis: cDNA was synthesized from the isolated RNA samples using the Transcriptor First Strand cDNA synthesis kit (Roche Diagnostics GmbH, Mannheim, Germany). For each subject, 1 μL ddH_2_O, 10 μL RNA, and 2 μL Random Primer were combined and incubated in Thermal Cycler for 10 min at 65°C. After incubation, 4 μL Reaction Buffer, 0.5 μL RNAase, 2 μL Deoxynucleotide Mix, and 0.5 μL Reverse Transcriptase were added; the reactions were incubated for 10 min at 25°C, 30 min at 55°C, 5 min at 85°C, then held at 4°C.

Quantitative gene expression evaluation by real-time polymerase chain reaction (RT-qPCR): For each cDNA sample, gene expression of both MPO and reference gene (G6PD) was analyzed using the Roche LightCycler 480 II Real-Time PCR instrument (Roche Diagnostics GmbH, Meinheim, Germany). PCR reactions in a final volume of 20 μL: 5 μL CDNA, 3 μL distiled water, 10 μL LightCycler 480 Probes Master (Roche Diagnostics GmbH, Mannheim, Germany), and 2 μL primer-probe set (Real-Time Ready single assay - Roche Diagnostics GmbH, Mannheim, Germany). Cycle conditions of the relative quantitative PCR (qPCR) were preincubated at 95°C for 10 min, followed by 45 amplification cycles of 95°C for 10 s, 6°C for 30 s, 72°C for 1 s, followed by cooling at 40°C for 30 s. qPCR analysis and calculation of quantification cycle (Cq) values for Relative Quantification were performed using the LightCycler 480 Software, Version 1.5 (Roche Diagnostics GmbH, Mannheim, Germany). Relative quantitative amounts were calculated by dividing the target genes by the expression level of the reference gene. Reference gene was used for normalization of target gene expression.

### Histopathologic examination

The cheek mucosa, lower lip, and tongue tissues removed from the animals were fixed in 10% formalin solution for 24 h. Following routine tissue processing, paraffin blocks were cut into 4 μm thick sections and stained with hematoxylin and eosin (H&E). All the sections were evaluated by a pathologist who was blinded to the treatment protocols under optic microscope (BX-52; Olympus, Tokyo, Japan).

### Statistical analysis

Experimental results were expressed as “mean±standard error” (x ±SEM). Significance of the difference between the groups was determined using one-way ANOVA test followed by LSD test. All statistical analyses were performed using the SPSS Statistics Version 22 statistical software, and p value <0.05 was considered significant.

## RESULTS

### Biochemical results

**Figure 1:**
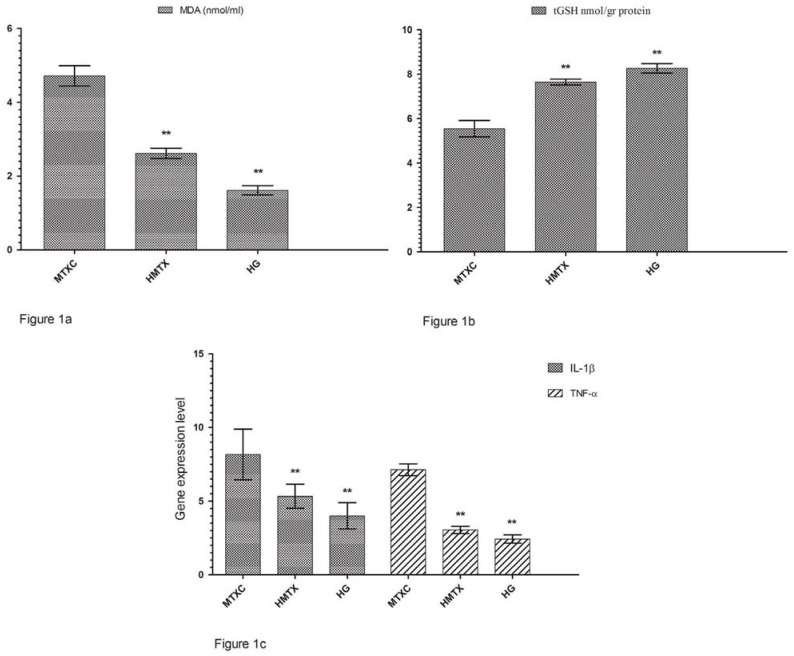
a: The effects of methotrexate on malondialdehyde (MDA) levels in the cheek mucosal tissues; b: The effects of methotrexate on tGSH levels in the cheek mucosal tissues; c: The effects of methotrexate on IL-1β gene expression level in the cheek mucosal tissues. *p<0.0001

**Figure 2:**
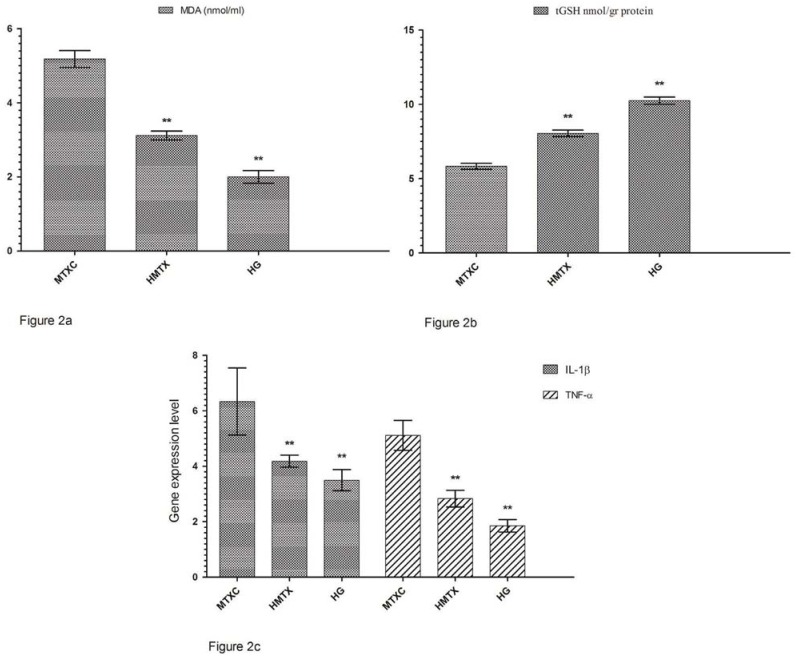
a: The effects of methotrexate on malondialdehyde (MDA) levels in the lower lip tissues; b: The effects of methotrexate on tGSH levels in the lower lip tissues; c: The effects of methotrexate on IL-1β gene expression level in the lower lip tissues. *p<0.0001

**Figure 3:**
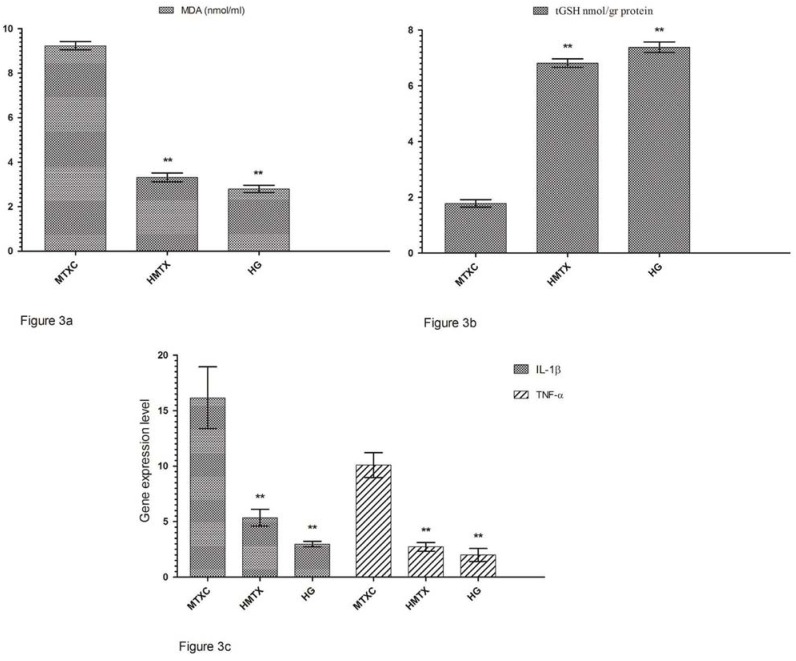
a: The effects of methotrexate on malondialdehyde (MDA) levels in the tongue tissues; b: The effects of methotrexate on tGSH levels in the tongue tissues; c: The effects of methotrexate on IL-1ß gene expression level in the tongue tissues. *p<0.0001

Figure 1a shows that the amounts of MDA were determined as 4.71±0.66, 2.61±0.34, and 1.61±0.30 μmol/gr protein, and the amounts of tGSH were found as 5.55±0.90, 7.65±0.32, and 8.26±0.51 nmol/gr protein in the cheek mucosal tissues of MTXC, HMTX, and HG groups; respectively (Figure 1b). MDA values were found as 5.18±0.55, 3.11±0.29, and 2.00±0.41 (Figure 2a) and, tGSH values were found as 5.83±0.49, 8.05±0.53, and 10.25±0.59 nmol/gr protein in the lower lip tissues of MTXC, HMTX, and HG groups; respectively (Figure 2b). The amounts of MDA were measured as 9.23±0.46, 3.31±0.47, and 2.80±0.40 μmol/gr protein in the tongue tissues of MTXC, HMTX, and HG groups (Figure 3a). Whereas, the amount of tGSH were found as 1.78±0.33, 6.81±0.37, and 7.38±0.46 nmol/gr protein in the tongue tissues of these groups (Figure 3b).

### Gene expression results

Orally administered MTX raised IL-1β gene expression in the cheek tissue of rats to 8.16±1.72. This rate was found as 5.33±0.81 and 4.00±0.89 in HMTX and HG groups. Again, MTX raised TNF-α gene expression in the cheek tissue of rats to 7.13±0.40, while HRE reduced TNF-α gene expression to 3.05±0.24. This rate was measured as 2.43±0.28 in HG group (Figure 1c). IL-1β gene expression in the lower lip tissue was found as 6.33±1.21, 4.18±0.21, and 3.50±0.38, and TNF-α gene expression was measured as 5.11±0.54, 2.83±0.30, and 1.85±0.22 in MTXC, HMTX, and HG, respectively (Figure 2c). Similarly, MTX significantly increased IL-1β and TNF-α gene expressions in the tongue tissue compared with HMTX and HG. IL-1β expression in the tongue tissue was found as 16.16±2.78, 5.35±0.76, and 2.98±0.24 and, TNF-α gene expression was measured as 10.11±1.12, 2.73±0.39, and 2.00±0.58 in MTXC, HMTX, and HG; respectively (Figure 3c).

### Histopathologic results

**Figure 4:**
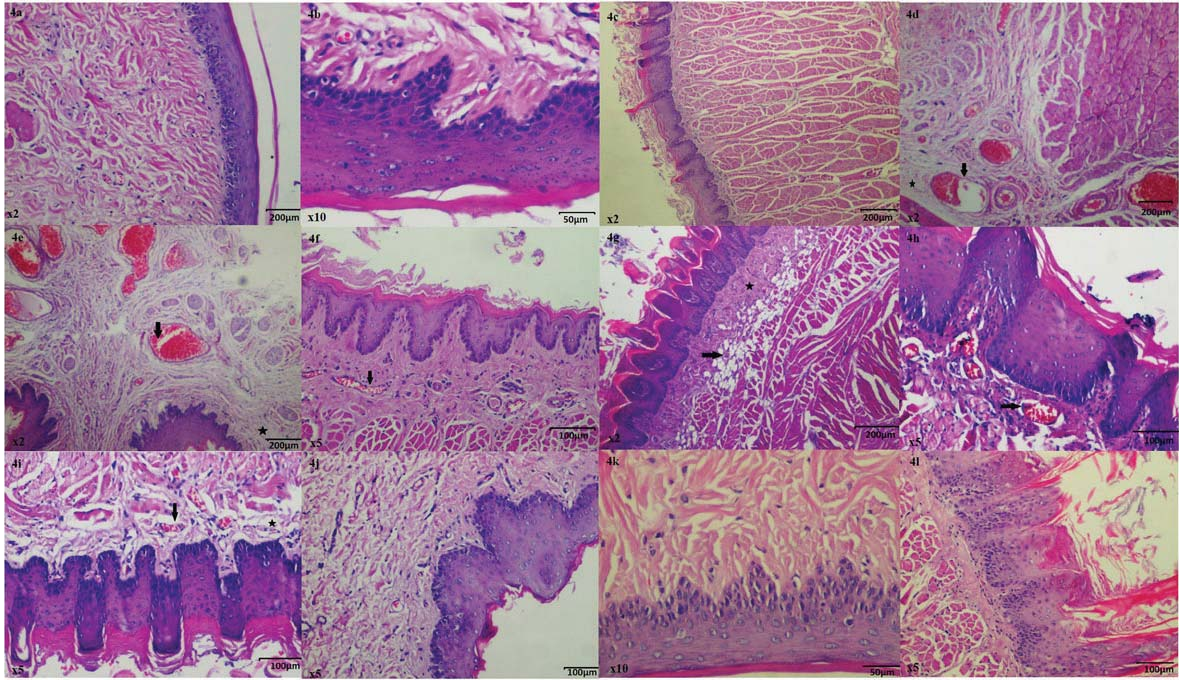
a: Normal histopathologic appearance of the rat cheek tissue of Healthy Group (HG) (HE&40); b: Normal histopathologic appearance of the rat cheek tissue of HG (HE&20); c: Normal histopathologic appearance of the rat cheek tissue of HG (HE&40); d: Dilated congested blood vessels (arrow) and edema (star) are distinguished in the cheek mucosa of MTXC group given methotrexate (MTX) (HEx40); e: Mild edema (star) and dilated congested blood vessels (arrow) are monitored in the lip mucosa of MTXC group given MTX (HEx40); f: Dilated congested blood vessels are seen in the tongue mucosa of MTXC group given MTX (HE&100); g: Fibroblastic proliferation (star) and fat cells replaced muscle layer are monitored in the tongue mucosal tissue of MTXC group given MTX (arrow) (HE&40); h: Dilated, congested, proliferated blood vessels (arrow) are seen in the tongue mucosa of MTXC group given MTX (HE&100); i: Edema (star) and dilated congested blood vessels (arrow) are monitored in the tongue mucosal tissue of MTXC group given MTX (HE&100); j: Appearance of the cheek tissue of HMTX group treated with *Hippophae rhamnoides* extract (HRE) resembling normal mucosa (HEx10); k: Appearance of the lip tissue of HMTX group treated with HRE resembling normal mucosa (HEx20); l: Appearance of the tongue tissue of HMTX group treated with HRE resembling normal mucosa (HEx100)

Examinations under optic microscope revealed a normal histopathologic appearance of the cheek, lower lip, and tongue tissues of the healthy group (Figure 4a, 4b, and 4c).

In Figure 4d, dilated congested blood vessels (arrow) and edema (star) were distinguished in the cheek mucosal tissue of MTXC group, which received MTX, while there were mild edema (star) and dilated congested blood vessels (arrow) observed also in the lower lip mucosa of this group (Figure 4e). However, besides dilated congested blood vessels (Figure 4f), fibroblastic proliferation (star) and fat cells replaced muscle layer (arrow) were also monitored in the tongue mucosal tissue of MTXC group (Figure 4g). Additionally, dilated congested proliferated blood vessels (Figure 4h) and marked edema (star) were found in the tongue tissue of MTXC group (Figure 4i). No pathologic finding was observed in the cheek, lower lip, and tongue tissues of the animals treated with HRE (Figure 4j, 4k, 4I).

## DISCUSSION

In this study, the effect of HRE on oral mucositis induced in rats with MTX was investigated and evaluated through biochemical, gene expression, and histopathologic examinations. Experimental results showed that the amount of MDA was increased and the amount of tGSH was decreased in the cheek, lower lip, and tongue tissues of the rats given MTX. Furthermore, IL-1β and TNF-α gene expressions were also significantly increased in the tissues in which MDA was significantly increased. MDA is known to be an oxidant and GSH an antioxidant parameter^17^. Therefore, increased MDA and decreased tGSH amounts indicate the development of oxidative stress. MTX has been experimentally demonstrated to increase the tissue levels of MDA, which is a marker of lipid peroxidation to decrease the level of tGSH^15,20^. HRE, which is used against the toxicity of MTX, was found to significantly prevent the increase of MDA and decrease of tGSH in the cheek, lower lip, and tongue tissues. This demonstrates that HRE protects the tissues against oxidative stress. Fruits of *Hippophae rhamnoides* contain numerous phytochemicals, such as vitamins A, E, C, carotens, fatty acids, and flavonoids^10,18,31^. These antioxidant phytochemicals are used to treat cancer, ulcer, liver, and skin diseases^32^. Many researchers have reported that lipid peroxidation products are increased, and the plasma level of vitamin E is decreased in the tissues of cancer patients due to chemotherapy^30^. Lipid peroxidation products increased with chemotherapy have been reported to decrease free radical scavengers antioxidant vitamins, such as vitamins A, E, and C^4,26^. In another *in vivo* and *in vitro* study, the use of vitamins E, A, and C against oxidative stress caused by chemotherapy has been found to enhance the therapeutic effect and also protect normal cells against apoptosis^5^. It has been reported in a cell culture and animal model that the combinations of vitamins A, B6, B12, C, D, and E with β-caroten protected against adverse effects of chemotherapy prolonged survival time and increased response to treatment^26^. Furthermore, it has been argued in a study evaluating risk-benefit ratio of β-caroten, vitamin E, vitamin C, and multivitamin combinations that vitamins had protective effect in cancer patients at risk^6^. This information from the literature indicates that our experimental results were in parallel with previous studies.

In this study, we also found that IL-1β and TNF-α gene expressions in the cheek, lower lip, and tongue tissues were significantly increased in the rats given MTX, compared with healthy and HRE groups. Chang CT noted that the levels of proinflammatory cytokines, such as IL-1β and TNF-α, were elevated in the oral tissue with mucositis developed due to chemotherapy^8^. Another study shows that the levels of IL-1β and TNF-α were increased and inflammatory ulcers were developed in the intestinal tissue with MTX^3^. In this study, IL-1β and TNF-α gene expressions were significantly increased in the tissues in which MDA was significantly increased. No information was found in the literature about the role of IL-1β and TNF-α in MTX oral mucositis. However, Çakir, et al.^7^ (2015) reported that MTX induced the amount of MDA and IL-1β and TNF-α gene expressions in the kidney tissue. In our experiment, HRE, which significantly prevented the increase of MDA, also significantly prevented the increase of IL-1β and TNF-α gene expressions. Vitamin C, alpha-tocopherol, and beta-carotens, also found in HRE, are known to have ihibitor effects on proinflammatory cytokines^23^. Again, palmitic, oleic, and linoleic fatty acids appear to exert antiinflammatory effects by inhibiting IL-1β and TNF-α^13^.

Macroscopically, no mucosal ulcerations were found in the cheek, lower lip, and tongue tissues of the animals given MTX. In addition, we found histopathological signs including mild edema and dilated congested blood vessels in the cheek and lower lip tissues, which have lower levels of MDA, IL-1β, and TNF-α than the tongue tissue. However, marked congested blood vessels, fibroblastic proliferation, fat cells replaced muscle layer, proliferated blood vessels, and edema were observed in the tongue mucosal tissue.

No pathologic findings were monitored in the cheek, lower lip, and tongue tissues of the healthy and HMTX groups, suggesting that histopathologic results were consistent with biochemical and gene expression results. Vascular congestion, inflammation, and fibroblastic proliferation are among the histopathologic findings of mucositis^11^. Nothing was found in the literature about excessive fat accumulation in the muscle layer due to MTX. However, several studies associate the accumulation of fat cells with inflammation and demonstrating this is a pathologic event^9^. Edema caused by MTX in the cheek, lower lip, and tongue tissue might be a result of vascular changes. In the literature, edema is one of the histopathologic findings accompanying vascular changes^24^. In conclusion, in this study we demonstrated with biochemical, gene expression, and histopathologic findings that MTX caused mild damage in the cheek and lower lip tissues and more severe damage in the tongue tissue of rats.

HRE was found to protect the cheek, lower lip, and tongue tissue against the toxic effect of MTX. These findings suggest that HRE may be useful for the prophylaxis of oral damage due to MTX.
